# Detecting Temporal Change in Dynamic Sounds: On the Role of Stimulus Duration, Speed, and Emotion

**DOI:** 10.3389/fpsyg.2015.02055

**Published:** 2016-01-13

**Authors:** Annett Schirmer, Nicolas Escoffier, Xiaoqin Cheng, Yenju Feng, Trevor B. Penney

**Affiliations:** ^1^Department of Psychology, National University of Singapore, SingaporeSingapore; ^2^Life Sciences Institute Programme in Neurobiology and Ageing, National University of Singapore, SingaporeSingapore; ^3^Duke-NUS Graduate Medical School, SingaporeSingapore; ^4^Graduate School for Integrative Sciences and Engineering, National University of Singapore, SingaporeSingapore

**Keywords:** auditory change detection, event-related potentials, vocal affect, sex differences, interval timing, preattentive, prosody

## Abstract

For dynamic sounds, such as vocal expressions, duration often varies alongside speed. Compared to longer sounds, shorter sounds unfold more quickly. Here, we asked whether listeners implicitly use this confound when representing temporal regularities in their environment. In addition, we explored the role of emotions in this process. Using a mismatch negativity (MMN) paradigm, we asked participants to watch a silent movie while passively listening to a stream of task-irrelevant sounds. In Experiment 1, one surprised and one neutral vocalization were compressed and stretched to create stimuli of 378 and 600 ms duration. Stimuli were presented in four blocks, two of which used surprised and two of which used neutral expressions. In one surprised and one neutral block, short and long stimuli served as standards and deviants, respectively. In the other two blocks, the assignment of standards and deviants was reversed. We observed a climbing MMN-like negativity shortly after deviant onset, which suggests that listeners implicitly track sound speed and detect speed changes. Additionally, this MMN-like effect emerged earlier and was larger for long than short deviants, suggesting greater sensitivity to duration increments or slowing down than to decrements or speeding up. Last, deviance detection was facilitated in surprised relative to neutral blocks, indicating that emotion enhances temporal processing. Experiment 2 was comparable to Experiment 1 with the exception that sounds were spectrally rotated to remove vocal emotional content. This abolished the emotional processing benefit, but preserved the other effects. Together, these results provide insights into listener sensitivity to sound speed and raise the possibility that speed biases duration judgements implicitly in a feed-forward manner. Moreover, this bias may be amplified for duration increments relative to decrements and within an emotional relative to a neutral stimulus context.

## Introduction

The human temporal sense depends on the ability to represent external events marking the passage of time. Research has shown that individuals encode such events outside awareness and automatically detect changes in event onset and duration (e.g., Näätänen et al., 1989; Tse and Penney, 2006). We asked whether individuals likewise track the speed with which events unfold and whether emotions benefit such tracking. Specifically, we explored brain responses to unattended neutral and emotional sounds that occasionally accelerated and decelerated, becoming shorter and longer as a consequence.

### Time Perception: On the Role of Stimulus Properties and Context

Time perception, a sixth human sense, critically contributes to meaningful interactions with the environment. Many mental functions depend on time. For example, attention is thought to be governed by temporal rhythms (Jones, 1976; Jones and Boltz, 1989; Escoffier et al., 2015). Learning in the context of classical and operant conditioning is shaped by the delay between two stimuli or between a behavior and its consequence (Gallistel and Gibbon, 2000). Language is temporally sensitive because the comprehension of words and syntactic dependencies relies on durational parameters such as voice-onset-time or intonation (Schirmer, 2004). Additionally, in the context of non-verbal communication, timing tells us how long to hold another’s gaze, to laugh at another’s joke, and to wait for another’s response before responding in turn.

Traditionally, the study of time perception has relied on simple static stimuli such as tones or images for which participants compared stimulus duration to a reference duration in memory. More recently, researchers have explored time perception with dynamic stimuli such as moving objects (Kaneko and Murakami, 2009; Matthews, 2011; Su and Jonikaitis, 2011; Linares and Gorea, 2015), tones (Matthews, 2013), music (Firmino et al., 2009; Droit-Volet et al., 2010; Cocenas-Silva et al., 2011; Darlow et al., 2013), faces (Fayolle and Droit-Volet, 2014), and vocalizations (Schirmer et al., in press). Although ecologically more valid, this approach presents a methodological challenge. With dynamic stimuli, duration always varies in conjunction with one or two other factors: stimulus content and speed. For example, compared to shorter vocalizations, longer vocalizations may contain more ups and downs in pitch (i.e., content varies), and/or the same pitch variation may play out more slowly (i.e., speed varies). At present, our understanding of how these two natural confounds impact time perception is incomplete.

Apart from using more ecologically valid materials, recent timing research has elucidated the role of contextual variables, such as emotion. For example, when asked to time the duration of a sound or image, temporal reproductions or duration judgements are typically longer for emotional as compared to neutral stimuli (e.g., Grommet et al., 2011; for reviews see Droit-Volet and Meck, 2007; Schirmer, 2011). Emotion effects differ depending on whether stimuli are static or dynamic and whether duration manipulations affect stimulus content as opposed to speed. Static timing stimuli tend to appear longer when they are emotional as compared to neutral (e.g., Grommet et al., 2011). The same is true for dynamic stimuli when duration manipulations are confounded by stimulus content, that is longer stimuli entail more events than do shorter stimuli (Angrilli et al., 1997). However, opposite effects emerge for dynamic stimuli that are confounded by speed, that is, longer stimuli play out more slowly than shorter stimuli (Voyer and Reuangrith, 2015; Schirmer et al., in press). Here, emotionality increases the probability of stimuli being perceived as short.

Although prior research has enhanced our understanding of the human temporal sense in the context of both static and dynamic events, this understanding is still incomplete. Moreover, the focus has been on explicit duration judgements and the representation of stimulus on- and offsets. Thus, we still know little about implicit timing and how individuals represent the temporal course of information between stimulus onset and offset. Additionally, it is unclear how such timing and temporal course representations are modulated by stimulus emotion.

### Mismatch Negativity: An Implicit Measure of Temporal and Emotional Processing

To address the issues outlined here, we adopted a mismatch negativity (MMN) paradigm. In this paradigm, participants pursue a primary activity while an auditory stimulus stream plays in the background. For example, they may watch a silent film while passively listening to a sequence of 440 Hz tones that is occasionally interrupted by a higher pitched deviant (Sams et al., 1985). The electroencephalogram (EEG), recorded throughout this procedure, reveals a negative event related potential (ERP) component peaking around 200 ms following stimulus onset with a fronto-central topography. This component, called the MMN, emerges when the average EEG response to standard stimuli is subtracted from the average EEG response to deviant stimuli. Over the mastoid electrodes, the MMN has positive polarity and this is thought to index generators in primary auditory cortex (for a recent review see Garrido et al., 2009).

The MMN has been used as a marker of implicit temporal processing in several studies (for a review see Ng and Penney, 2014). Stimuli comprised sequences of tones, vowels, syllables or music and stimuli within a sequence differed in duration only. For example, Jacobsen and Schröger (2003) presented several experimental blocks, one of which comprised frequent standard tones of 150 ms and rare deviant tones of 100 ms. Similar to others, these authors found that temporal deviants elicited an MMN peaking around 250 ms following sound onset. In case of shorter deviants, as was true for their block, temporal change occurs at deviant offset. In case of longer deviants, temporal change occurs during the deviant (i.e., at the value of the standard duration). Notably, there is evidence that MMN amplitude differs between these cases (but see Amenedo and Escera, 2000; Peter et al., 2012), although some studies found that long deviants produce a larger MMN than short deviants (e.g., Catts et al., 1995; Jaramillo et al., 1999; Atkinson et al., 2012), whereas other studies found the opposite (e.g., Jaramillo et al., 1999; Takegata et al., 2008; Colin et al., 2009). Why change direction matters and why its influence varies are still open questions and will be considered further in the discussion of this paper.

Apart from being sensitive to temporal change, the MMN is also sensitive to emotional change. In a first study, Schirmer et al. (2005) presented listeners with the pseudoword “dada” spoken in a neutral and angry voice. In some blocks, neutral sounds served as standards and angry sounds as deviants, whereas in other blocks, angry sounds served as standards and neutral sounds as deviants. A separate experiment used neutral and happy voices. Both emotional sounds elicited an earlier and larger MMN than the neutral sounds, indicating that emotions facilitated change detection. Moreover, sex differences in this effect indicated that women were more sensitive than men to unattended emotional expressions. Subsequent research replicated these results (Schirmer et al., 2007; Schirmer and Escoffier, 2010; Thönnessen et al., 2010; Fan et al., 2013) and pointed to sources in the temporal lobe and insula (Schirmer et al., 2008; Thönnessen et al., 2010).

### The Present Study

Clearly, the MMN can be used to study implicit temporal processing. However, existing MMN studies focused on duration effects, that is whether a deviant expires before or after a standard, rather than on dynamic time course effects that emerge throughout a stimulus. Moreover, the role of emotion, which is known to affect both timing and the MMN, remains to be explored. Here, we sought to address these gaps. We created MMN stimuli by subjecting one surprised and one neutrally spoken “Ah” to a speech manipulation procedure creating a 378 ms and a 600 ms exemplar, of which the former had a fast and the latter a slow speed. We then presented these exemplars in four blocks, two of which comprised surprised and two of which comprised neutral stimuli. Within each emotion condition, one block used the short exemplar as the standard and the long exemplar as the deviant, whereas the other block had a reversed stimulus assignment. Participants passively listened to the four blocks, while watching a silent subtitled movie.

In line with previous research using static stimuli, we expected a duration MMN peaking about 200 ms after deviant offset (short deviant blocks) or the duration value of the standard (long deviant blocks). Additionally, we expected ERP differences between deviants and standards due to the dynamic nature of our stimuli. Specifically, if listeners implicitly process the slowing down and speeding up associated with long and short deviants, respectively, the deviant ERP should become more negative than the standard ERP prior to the stimulus duration mismatch. Moreover, we anticipated an incremental mismatch effect emerging shortly after stimulus onset as information about temporal change continuously accumulated and the temporal disparity between standard and deviant increased. Last, we hypothesized that emotions would facilitate temporal change detection resulting in an earlier and larger mismatch effect for the surprised relative to the neutral blocks, especially for female listeners.

## Experiment 1

### Methods

This research was approved by the Institutional Review Board of the National University of Singapore.

#### Participants

We recruited 35 participants, most of whom were students, through campus advertisements. The data from three participants were discarded due to excessive movement artifacts in the EEG. Half of the remaining participants were male and the other half were female. The average age was 22.2 (*SD* = 2.4). Participants reported normal hearing and normal or corrected to normal vision. They were reimbursed for their time at a rate of S$10/hour.

#### Stimuli

The stimulus material consisted of the interjection “Ah” spoken in a neutral and a surprised voice by a male speaker. The recordings were taken from a larger pool of 231 interjections produced by 33 speakers and including neutral, surprised, sad, angry, happy, disgusted, and fearful expressions. These stimuli were presented to 30 individuals (18 female and 12 male, mean age = 22.1, *SD* = 2.2) who did not participate in the main experiment and who indicated whether a given stimulus expressed neutrality, surprise, sadness, anger, happiness, disgust, fear or another self-determined emotion and rated stimulus arousal on a 5-point scale ranging from 1 (very calm) to 5 (very excited). Recognition probability (i.e., number of raters who correctly identified the emotion divided by the total number of raters) and mean arousal were 0.97 and 2.4 (*SD* = 1.09) for the selected neutral stimulus and 0.7 and 3.13 (SD = 0.78) for the selected surprised stimulus. The neutral stimulus had a duration of 501 ms, whereas the surprised stimulus had a duration of 502 ms.

**FIGURE 1:**
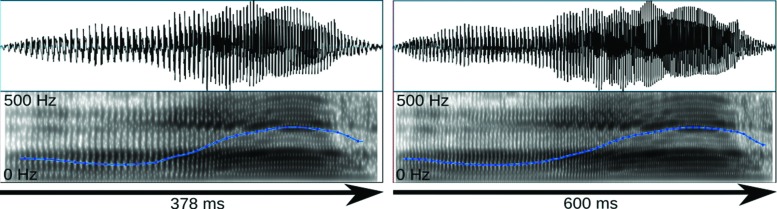
**Oscillogram (top) and spectrogram (bottom) for the short and long exemplars of the surprised stimulus.** Blue lines reflect the fundamental frequency contour heard as pitch.

The stimuli were selected based on their rating results and their suitability for the compression/stretching manipulation employed here. Specifically, interjections were manipulated with Celemony Melodyne 2, a commercial sound manipulation software that implements an algorithm for duration change. According to the developer information, this algorithm was designed to enable duration change without altering average pitch and short-term spectral features. Looking at mean pitch and pitch variation we could confirm this claim for a larger stimulus set presented elsewhere (Schirmer et al., in press). However, the algorithm appears to affect harmonics-to-noise ratio (HNR), reducing it in the case of sound compression and increasing it in the case of sound stretching. Nevertheless, we subjected the stimuli described above to the algorithm resulting in a short version of 378 ms and a long version of 600 ms (**Figure 1**). The short and long versions differed in HNR by 2.5 dB only. Sound amplitudes were normalized in MATLAB to the same root-mean-square (RMS) value.

#### Paradigm

**FIGURE 2:**
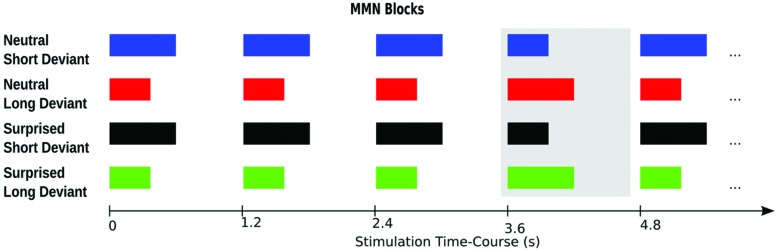
**Experimental paradigm.** The four experimental blocks are illustrated in rows one to four, respectively. The deviant in each block is highlighted by the gray box. Each participant completed all four blocks in counterbalanced order.

For an illustration of the paradigm please see **Figure 2**. The sounds were presented in four blocks, each comprising 630 standards and 105 deviants. Two blocks used surprised expressions and the other two blocks used neutral expressions. One surprised and one neutral expression block used the short stimulus as the standard and the long stimulus as the deviant. The other two blocks used the long stimulus as the standard and the short stimulus as the deviant. Deviants were presented pseudorandomly such that they followed a sequence of 3–9 standards. Stimulus onset asynchrony was kept constant at 1.2 s. Block order was counterbalanced across participants using a Latin Square design.

The stimuli were played over ear-insert headphones at a comfortable sound pressure level that was kept constant across participants. While listening to the sounds, participants watched a self-selected, silent, subtitled movie. They were offered nine movies to chose from, which had an average duration of 109 min (range 94–134) and were classified by the British Board of Film as 15, 12a or appropriate for universal audiences. The authors of this research considered the movies to be only mildly arousing as they excluded comedies, dramas, action or extremely violent movies. The experiment lasted about an hour.

#### Electrophysiological Recording and Analysis

The EEG was recorded using a 24-channel ANT system at a sampling rate of 256 Hz. Electrodes were placed according to the modified 10–20 system. An online anti-aliasing filter was applied with a cut-off frequency at 0.27 times the sampling rate.

The data were processed in EEGLAB. Continuous recordings were epoched and baseline-corrected using a 200 ms window prior to stimulus onset and an 800 ms window following stimulus onset. After low- (30 Hz cutoff, 6.75 Hz transition band) and high-pass filtering (0.1 Hz cutoff, 0.2 Hz transition band), the data were scanned visually for non-typical artifacts and subjected to Infomax, an independent components algorithm. Components reflecting eye-blinks and saccades were removed, and the back-projected data scanned once more for residual artifacts.

For statistical analysis, we focused on nine frontal and central channels where the MMN is typically observed (i.e., FP1, F3, C3, Fpz, Fz, Cz, FP2, F4, C4). We included all deviant trials as well as standards immediately preceding a deviant that had survived artifact rejection. Visual inspection of the data failed to reveal a sharp MMN component. Instead, we observed a ramping negativity that developed throughout the stimulus epoch. To adequately capture this negativity, we divided stimulus epochs into eight 100 ms windows and subjected the mean voltages from these windows to an ANOVA with *Duration* (short/long), *Emotion* (neutral/surprised), *Deviance* (standard/deviant), and *Window* (1–8) as repeated measures factors and *Sex* as a between subjects factor. Due to our interest in temporal change detection, we focus this report on main effects and interactions involving *Deviance*.

### Results

**FIGURE 3:**
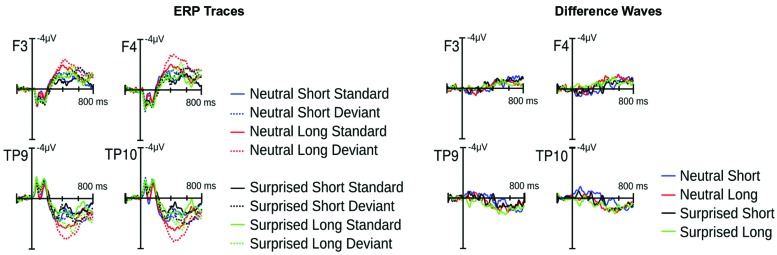
**Event related potential (ERP) results from Experiment 1.** Illustrated on the left are condition traces for two frontal channels of the left (F3) and right (F4) hemisphere and for the two mastoid channels. Illustrated on the right are differences waves computed by subtracting short standards from short deviants and long standards from long deviants.

**FIGURE 4:**
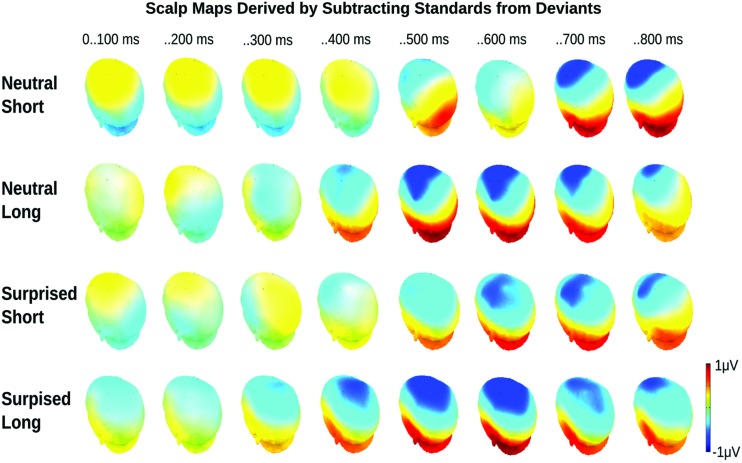
**Event related potential topographical maps for Experiment 1.** Maps present the mean potential differences between standards and deviants for the eight 100 ms analysis windows.

Visual inspection of the ERPs revealed a climbing negativity, in some cases preceded by a positive dip, over fronto-central electrodes with polarity inversion over the mastoids. The negativity developed earlier and was larger for long relative to short deviants and within the surprised relative to the neutral vocal stream (**Figures 3** and **4**). Effects seemed comparable for female and male listeners.

Statistical analysis confirmed these visual impressions. It revealed a significant interaction between *Deviance* and *Window* [*F*(7,210) = 46.5, *p* < 0.0001] compatible with a growing difference between standards and deviants throughout the course of the stimulus. Additionally, the *Window* × *Deviance* × *Duration* [*F*(7,210) = 6.9, *p* < 0.0001] and the *Window* × *Deviance* × *Emotion* [*F*(7,210) = 3.35, *p* < 0.01] interactions were significant. Hence, we pursued these effects for each time bin separately (compare time bin maps illustrated in **Figure 4**).

The *Deviance* × *Duration* interaction was significant for *Windows* 1, 3, 4, 5, and 6 [*F*s(1,30) > 7, *p*s < .013]. In the first window, short deviants elicited a more positive ERP than short standards [*F*(1,30) = 13.8, *p* < 0.001], but long deviants and standards did not differ (*p* > 0.1). In the third and fourth windows, long deviants elicited a more negative ERP than long standards [*F*s(1,30) > 7, *p*s < 0.013], but short deviants and standards did not differ (*ps* > 0.1). In the fifth and sixth windows, the ERPs for both long [*F*s(1,30) > 41, *p*s < 0.0001] and short duration stimuli [*F*s(1,30) > 11, *p*s < 0.003] were more negative for deviants than standards.

The *Deviance* × *Emotion* interaction was significant for Windows 1, 2, and 3 [*F*s(1,30) > 6.5, *p*s < 0.016]. In the first and second windows, neutral deviants elicited a more positive ERP than neutral standards [*F*s(1,30) > 13.7, *p*s < 0.001], whereas surprised deviants and standards did not differ (*p*s > 0.1). In the third window, surprised deviants elicited a more negative ERP than surprised standards [*F*(1,30) = 8.9, *p* < 0.01], whereas neutral deviants and standards did not differ (*p* > 0.1).

In windows 7 and 8, only the *Deviance* main effect reached significance [*F*s(1,30) = 44, *p* < 0.0001]; irrespective of *Duration* and *Emotion*, deviants elicited a more negative ERP than standards.

### Discussion

Experiment 1 explored whether listeners perceive changes in the acoustic rate of unattended vocalizations and whether this perception is facilitated within an emotional as compared to a neutral auditory context. Our results support both propositions. Although the typical MMN component was absent, we found a climbing negativity for both long and short deviants with MMN-like topography and polarity inversion over the mastoids. Moreover, in the case of long deviants, this negativity emerged at around 200 ms and thus 178 ms before the duration value of short standards. This indicates that listeners were sensitive to a slow-down in stimulus speed. There was also a climbing negativity for short deviants. This negativity developed around 400 ms, thus, roughly coinciding with deviant offset and preceding the long standard offset time by 200 ms. Note that a mismatch response driven entirely by duration should manifest approximately 200 ms after deviant offset. So, although later and smaller than the effect for long deviants, the short deviant effect suggests that listeners perceive an unattended speed-up.

Emotions modulated the emergence of temporal deviant effects in the ERP. The climbing negativity appeared about 100 ms earlier in the surprised relative to the neutral stimulus stream, regardless of deviant duration. Notably, this effect showed irrespective of listener sex suggesting that men and women were equally sensitive to the vocal emotional context.

In the following section, we report a second experiment aimed at pursuing these results further. Specifically, by presenting spectrally rotated versions of the sounds used in Experiment 1, we intended to remove obviously human vocal features from the stimuli, while preserving temporal and spectral stimulus complexity (Blesser, 1972; Scott et al., 2000; Warren et al., 2006; Christmann et al., 2014). The sounds were created by flipping the frequency spectrum around a central frequency resulting in an “alien” sound quality. Thus, we hoped to answer two questions. First, we were interested in determining whether the time course of temporal change detection depends on the presentation of socially relevant vocal expressions as compared with non-vocal sounds. In other words, are differences in sensitivity to a slow-down and speed-up in acoustic rate modulated by whether the sound has human qualities? Second, we wished to determine whether emotion effects in Experiment 1 were due to affective or acoustic stimulus characteristics. Perhaps sound idiosyncrasies rather than their emotional meaning affected the time course of temporal change detection.

## Experiment 2

### Methods

This research was approved by the Institutional Review Board of the National University of Singapore.

#### Participants

We recruited 33 participants. The data from one participant were discarded due to excessive artifacts in the EEG. Half of the remaining participants were male and the other half were female. Their average age was 22.5 (*SD* = 2.3). Participants reported normal hearing and normal or corrected to normal vision. They were reimbursed for their time at a rate of S$10/hour.

#### Stimuli

**FIGURE 5:**
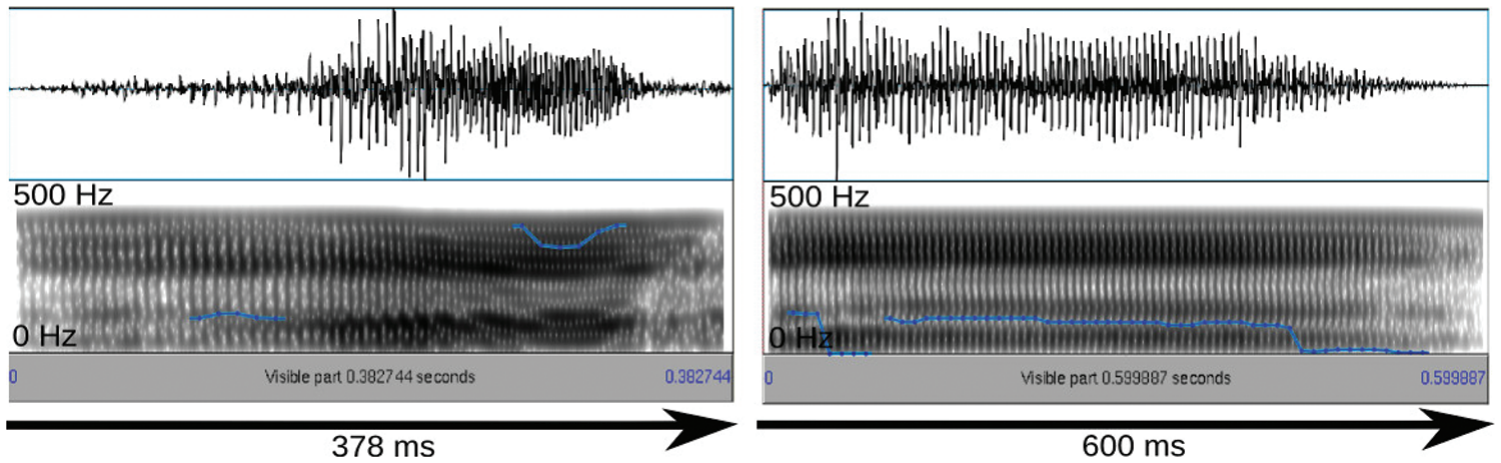
**Oscillogram (top) and spectrogram (bottom) for the short and long exemplars of the spectrally rotated surprised stimulus.** Blue lines reflect the fundamental frequency contour heard as pitch.

The interjections from Experiment 1 were low-pass filtered (3.8 kHz) and subjected to a spectral rotation around 2 kHz (Blesser, 1972; Scott et al., 2000). Sound amplitudes were normalized in MATLAB to the same RMS value. The sounds are illustrated in **Figure 5**.

#### Paradigm

The paradigm was the same as in Experiment 1.

#### Electrophysiological Recording and Analysis

Data recording and processing were the same as in Experiment 1.

### Results

**FIGURE 6:**
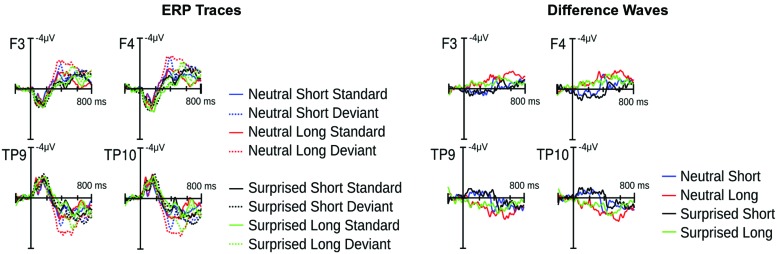
**Event related potential results from Experiment 2.** Condition traces for two frontal channels of the left (F3) and right (F4) hemisphere and the two mastoid channels are illustrated on the left. Differences waves computed by subtracting short standards from short deviants and long standards from long deviants are illustrated on the right.

**FIGURE 7:**
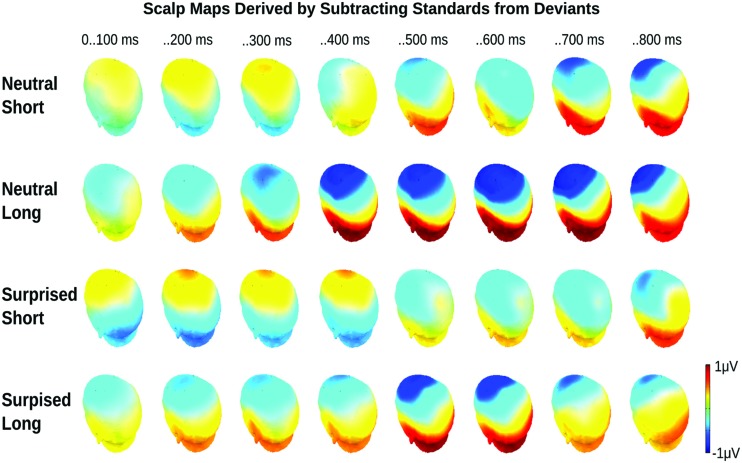
**Event related potential topographical maps for Experiment 2.** Maps present the mean potential differences between standards and deviants for the eight 100 ms analysis windows.

Visual inspection of the ERP suggested similarities with and differences from Experiment 1. There was again a climbing fronto-central negativity with polarity inversion over the mastoids for deviants relative to standards. Again, in some cases, this negativity was preceded by a positive dip. Moreover, long deviants produced an earlier and larger negativity than short deviants. However, differences between surprised and neutral vocalizations appeared reversed relative to Experiment 1 (**Figures 6** and **7**).

To probe these visual impressions, we subjected mean voltages from eight 100 ms windows to an ANOVA with *Duration*, *Emotion*, *Deviance*, and *Window* as repeated measures factors and *Sex* as a between subjects factor. This analysis revealed a significant *Deviance* × *Window* interaction [*F*(7,210) = 35.4, *p* < 0.0001] compatible with a growing difference between standards and deviants throughout the course of the stimulus. Additionally, the *Window* × *Deviance* × *Duration* [*F*(7,210) = 9.4, *p* < 0.0001], the *Window* × *Deviance* × *Emotion* [*F*(7,210) = 5.3, *p* < 0.0001], and the *Window* × *Deviance* × *Duration* × *Emotion* [*F*(7,210) = 2.1, *p* < 0.05] interactions were significant. Hence, we pursued these effects for each time bin separately (compare maps presented in **Figure 7**).

The *Deviance* × *Duration* × *Emotion* interaction was significant in the first analysis window only [*F*(1,30) = 4.5, *p* < 0.05; other *p*s > 0.1]. A follow-up analysis for long durations revealed an effect of *Deviance* only [*F*(1,30) = 13.5, *p* < 0.001]. Long deviants elicited a more negative ERP than long standards. A follow-up analysis for short durations revealed a *Deviance* × *Emotion* interaction [*F*(1,30) = 5.6, *p* < 0.05] indicating that the *Deviance* effect was significant for surprised [*F*(1,30) = 29.9, *p* < 0.0001], but not neutral expressions (*p* > 0.1). Over the first 100 ms following stimulus onset, surprised deviants elicited a more positive ERP than surprised standards.

The *Deviance* × *Duration* interaction was significant in windows 2, 3, 4, 5, 6, and 7 [*F*s(1,30) > 6.7, *p*s < 0.05]. In windows 2, 3, and 4, the ERP was more positive for short deviants than for short standards [*F*s(1,30) = 36, 24, *p*s < 0.0001; *F*(1,30) = 3.4, *p* = 0.07] and more negative for long deviants than for long standards [*F*s(1,30) > 25, *p*s < 0.0001]. Subsequently, both short [*F*s(1,30) > 9, *p*s < 0.01] and long [*F*s(1,30) > 42, *p*s < 0.0001] deviants elicited more negative potentials than standards. However, the effect for long durations was greater.

The *Deviance* × *Emotion* interaction was significant in windows 4 and 7 [*F*s(1,30) > 10, *p*s < 0.01]. In window 4, the deviant ERP was more negative than the standard ERP in the neutral [*F*(1,30) = 30.7, *p* < 0.0001], but not the surprised condition (*p* > 0.1). In window 7, the deviant ERP was more negative than the standard ERP in both conditions, but this difference was larger for the neutral [*F*(1,30) = 49.2, *p* < 0.0001] than the surprised [*F*(1,30) = 10.5, *p* < 0.01] condition.

In window 8, only the *Deviance* main effect reached significance [*F*(1,30) = 45.5, *p* < 0.0001]. Irrespective of *Duration* and *Emotion*, deviants elicited a more negative ERP than standards.

## General Discussion

The present study explored the implicit processing of temporal change within emotional and neutral streams of dynamic stimuli. Using an MMN paradigm, we found evidence that listeners mentally represent task-irrelevant increments and decrements in stimulus speed and that their representations differ as a function of emotion. In the following, we highlight the contributions of these results to the literature on (1) dynamic timing, (2) the asymmetry of temporal change effects, as well as (3) the role of emotions for time perception.

### Temporal Processing of Dynamic Events

To our knowledge, this is the first demonstration that listeners mentally represent, not only duration, but also the speed with which task-irrelevant events unfold. More negative ERPs for deviants than standards emerged shortly after deviant onset and, thus, before the standard or deviant duration had lapsed. This points to an immediate sensitivity to the rate at which auditory neurons are excited. Furthermore, it suggests that an increasing disparity between standard and deviant time course contributes incrementally to an emerging representation of temporal change. We speculate that this representation then automatically biases an individual’s perception of stimulus duration.

Explicit timing research accords with this speculation. Participants asked to judge the duration of dynamic stimuli demonstrate temporal distortions pointing to an influence of stimulus content or speed (Eagleman, 2008; Matthews, 2011; Liverence and Scholl, 2012; Linares and Gorea, 2015). For example, a greater frequency of loops (i.e., change in content) made by a luminance blob was associated with a lengthening of subjective duration (Linares and Gorea, 2015). Furthermore, faster changes in vocal pitch (i.e., change in speed) were associated with a shortening of subjective duration (Schirmer et al., in press). Our results add to this literature by addressing implicit timing and by showing that representations of stimulus speed emerge incrementally throughout a stimulus and could, hence, contribute to duration perception in a feed-forward manner.

The present auditory change effect differed somewhat from the auditory change effect seen in previous MMN studies (for reviews see Näätänen et al., 2005; Garrido et al., 2009). Previous reports described a negative component with a fronto-central topography, that inverts polarity over the mastoids and peaks about 200 ms following deviant onset. Our effects match these properties with the exception that there was no clearly defined component peak. Instead, we observed a climbing negativity that plateaued later in the epoch. Nevertheless, we suspect this negativity to be an MMN and to index auditory change detection. Differences in time course or amplitude contour probably arise from the nature of our manipulation, which produced an incremental rather than a sudden difference between standards and deviants.

Notably, the MMN effect observed here resembles a negative ERP deflection often reported in the timing literature. This deflection, called the contingent negative variation (CNV), is maximal over fronto-central leads and was shown by some to increase in amplitude with increasing stimulus duration (Macar and Vitton, 1980). As such it was thought to reflect temporal encoding (Macar and Vidal, 2003). More recently, however, the CNV is deemed more likely to be an indicator of response preparation or temporal decision-making (Kononowicz and van Rijn, 2011; Ng et al., 2011; Van Rijn et al., 2011). Given the resemblance of CNV and the present temporal change effect one may wonder whether these are distinct or overlapping phenomena. We suggest that they are distinct based on effect topography and eliciting conditions. The present climbing negativity, but not the CNV (Ng and Penney, 2014), has sources in the primary auditory cortex as indicated by ERP inversion over mastoid electrodes. Additionally, one would expect a CNV like effect in an MMN paradigm for both standards and deviants with amplitude differences emerging only after the point of duration mismatch. The effects observed here, however, were present before this point.

### Asymmetry in the Sensitivity to Temporal Change

Existing research indicates that listeners differ in their sensitivity to duration decrements and increments (but see Amenedo and Escera, 2000; Peter et al., 2012). Some studies have found a larger MMN to short than to long deviants (Jaramillo et al., 1999; Colin et al., 2009), whereas others (Catts et al., 1995; Takegata et al., 2008; Atkinson et al., 2012), including the present study, have found the opposite. Three factors were cited to explain this variation. First, asymmetry in the sensitivity to temporal change may depend on stimulus properties (Jaramillo et al., 1999; Takegata et al., 2008). For example, Jaramillo et al. (1999) found a larger MMN to short than to long vowels, but a smaller MMN to short than to long tones pointing to processing differences between vocal and non-vocal sounds. Notably, the present study conflicts with this result. The MMN to both interjections (Experiment 1) and their spectrally rotated counterparts (Experiment 2) was greater in the long relative to the short condition.

A second explanation of the asymmetry in temporal change detection invokes a role of absolute stimulus duration. For stimuli that are less than 200 ms long, an increase in duration is perceived as an increase in sound intensity (Takegata et al., 2008). Thus, a 150 ms deviant may be perceived as louder than a preceding 100 ms standard, whereas a 100 ms deviant may be perceived as softer than a preceding 150 ms standard. At these durations, then, differences in MMN magnitude may result from an asymmetry in the perception of sound intensity rather than duration (Peter et al., 2010). For stimuli that exceed 200 ms, perceived sound intensity does not differ as a function of duration. In the present study, stimuli were 378 and 600 ms long, so an intensity illusion is unlikely to account for the observed MMN effects.

Last, asymmetry in temporal change detection has been linked to the method by which an MMN is generated. Traditionally, researchers subtracted standards from deviants in the same block. More recently, however, approaches controlling for the physical difference between standards and deviants have become popular (Jacobsen and Schröger, 2003). One approach involves an additional experimental block in which deviants are presented in an equiprobable manner together with other stimuli. Another approach involves an additional experimental block in which the role of standards and deviants is reversed. In either case, an MMN is generated by subtracting the physically identical control stimulus from the deviant. A comparison of the traditional with the physical control approach indicated that MMN asymmetries for duration deviants are present in the former, but not the latter (Peter et al., 2010). Again, this explanation does not account for our results as we used the physical control approach, but nevertheless observed differences in the MMN to short and long deviants.

Together, the literature on MMN asymmetry is inconclusive. Although asymmetry is frequently observed, it seems to depend on a number of stimulus parameters. Moreover, these parameters may include two methodological novelties implemented in the present study. Our stimulus durations were fairly long and we manipulated duration alongside speed. Both of these factors may explain why our results are similar to some studies, but differ from others.

Although we are unable to explain variation in MMN asymmetry, we can offer an explanation for why the MMN was larger for increments here. Specifically, behavioral research on timing suggests that repeated or expected stimuli become subjectively shorter than non-repeated or unexpected stimuli, respectively (Pariyadath and Eagleman, 2007; Matthews et al., 2014; for a possible dissociation between repetition and expectation see Matthews, 2015). Moreover, because temporal lengthening and shortening are typically associated with a speed increase and decrease in a dynamic context, these illusions may extend to the perception of speed. Repeated and/or expected events may appear faster than unrepeated and/or unexpected events. Thus, in the present MMN paradigm, long and short deviants might have appeared more and less different from their standard, respectively, and this asymmetry probably emerged as speed discrepancies accumulated.

Before closing the discussion of MMN asymmetry, we would like to add a caveat that complicates the interpretation of duration effects. Likely, here and elsewhere, duration deviants not only violated the expectation for a particular stimulus duration, but also distorted an overall rhythmic structure established by standards (Jones, 1976; Escoffier et al., 2010). Blocks, although comparable in stimulus onset timing, differed in standard duration and speed possibly creating different emphases or metric points that then modulated stimulus processing. As illustrated in **Figure 2**, the longer standards may have been metrically more important than the short standards, thus, producing a stronger entrainment and more readily accommodating temporal deviants. Such a modulation would be revealed by an interaction between stimulus deviance (standard/deviant) and duration (short/long), whereby the deviance effect would be reversed for the short and long duration conditions. In other words, there would be a block effect whereby short standards and long deviants presented together in one block would differ in comparable ways from long standards and short deviants presented together in another block.

Statistical analysis of the present data revealed patterns suggesting such block effects. In Experiment 1 (vocal), initially, short deviants elicited a more positive potential than short standards, whereas the deviance effect for long stimuli was non-significant. In Experiment 2 (non-vocal), initially, short deviants elicited a more positive potential, while long deviants elicited a more negative potential relative to their respective standards. Thus, rhythmic processing likely occurred alongside duration processing and affected the ERP. The present as well as previous studies cannot dissociate the two. To achieve this, future research could compare temporally regular with irregular stimulus presentations (McAuley and Fromboluti, 2014).

### Emotions and Temporal Change Detection

A final objective of this study was to determine whether and how emotions influence temporal change detection. As expected, we found an earlier change effect for surprised relative to neutral vocalizations. This latency difference reversed when vocalizations were spectrally rotated and human expressiveness was removed (Warren et al., 2006). Moreover, while the time course of ERP effects was comparable for neutral original and rotated sounds, that of surprised original and rotated sounds differed. Additionally, the amplitude of the MMN-like effect in neutral and surprised conditions was comparable for original vocalizations, whereas the same effect was larger for the neutral than the surprised condition for spectrally rotated sounds. Together, these observations suggest that vocal emotions overwrote the processing differences that were due to non-emotional stimulus properties (e.g., stimulus complexity) and facilitated the neural representation of temporal change.

The present emotion effect concurs with prior research demonstrating emotion effects on the MMN and on performance in temporal judgment tasks. As reviewed in the introduction, emotional deviants following neutral standards elicit an earlier and larger MMN than neutral deviants following emotional standards (e.g., Schirmer et al., 2005). Additionally, stimuli with emotional valence are perceived as longer than same-duration stimuli with neutral valence (e.g., Grommet et al., 2011). Together these and the present results suggest that emotions enhance the salience of a stimulus stream ensuring sufficient processing resources despite being task-irrelevant.

This proposition is in line with recent evidence for a role of attention in the link between emotions and time. Rather than asking participants to time emotional and neutral stimuli, Lui et al. (2011) manipulated the emotionality of distractors presented while participants timed simple visual shapes. Specifically, an initial circle (S1) was followed by a distractor and then a second circle (S2) and participants decided whether S2 was shorter or longer than S1. Participants were more likely to judge S2 to be shorter than S1 if the distractor was emotional as compared to neutral. This suggests that S2 indeed seemed shorter in the emotional as compared to neutral context. Moreover, it implies that emotional stimuli capture and bind processing resources at the cost of other neutral stimuli (for similar approaches and results see Halbertsma and Van Rijn, in press; Lake et al., in press).

Change detection research accords with this. Apart from the present study, there is one previous attempt at comparing the MMN in an emotional and neutral context. Lv et al. (2011) asked participants to indicate whether two faces presented side-by-side were identical. In different blocks, faces had a sad or neutral expression. In the background, participants heard a sequence of tones that contained rare deviants. Unlike in the present study, the MMN was comparable in the emotional and neutral blocks. However, face discrimination was facilitated by sad relative to neutral expressions. Thus, emotions effectively held attention to the visual material thereby benefiting visual categorization performance rather than the processing of an unrelated stream of neutral sounds.

Although in agreement with existing work, the present study failed to identify sex differences in the emotion effect. Temporal change detection was enhanced for surprised relative to neutral vocal streams in both men and women. Given earlier work (Fan et al., 2013; Schirmer et al., 2005, in press), this result was somewhat unexpected. We suspect that the absence of sex effects here relates to the fact that emotions varied in a blocked rather than an event-related manner. There is some indication that sex differences in vocal emotion sensitivity are a matter of processing time and attention. In an implicit emotional priming paradigm, only women showed priming at a short (200 ms) prime-target interval, whereas both sexes showed priming at a longer (750 ms) prime-target interval (Schirmer et al., 2002). Moreover, when emotions were made task-relevant by asking participants to categorize stimuli based on emotion, sex differences likewise disappeared (Schirmer et al., 2006, 2013). Thus, we speculate that the repetition of vocal expressions within blocks enabled participants to “tune into” a particular emotion condition putting male and female processing on par.

## Conclusion

For complex environmental stimuli, stimulus duration is necessarily confounded by content and/or speed. Here, we made a first attempt at assessing combined duration and speed perception without highlighting temporal processing to our participants. In the context of an MMN paradigm, we observed a climbing MMN-like negativity emerging shortly after deviant onset. Thus, we conclude that stimulus speed is tracked continuously and suggest that it biases duration perception well before stimulus offset in a feed-forward manner. In the present study, like some others, mismatch effects showed earlier and were larger to duration increments relative to decrements. Although the factors underpinning this are still unclear, we suspect a role of stimulus predictability. Contrasting repeated standards with singular deviants can be expected to augment and diminish perceived temporal change for long and short deviants, respectively. As was demonstrated before, emotions influenced temporal processing. Change was detected earlier within a stream of surprised relative to neutral vocalizations suggesting that the former recruit more processing resources than the latter.

## Author Contributions

AS was involved in study design, data analysis, and manuscript writing. NE was involved in study design, experimental programming, data acquisition and analysis. He also commented on the manuscript. XC and YF were involved in data acquisition and commented on the manuscript. TP was involved in study design and manuscript writing.

## Conflict of Interest Statement

The authors declare that the research was conducted in the absence of any commercial or financial relationships that could be construed as a potential conflict of interest.
